# Prevalence of Long COVID-19 Symptoms After Hospital Discharge in Frail and Robust Patients

**DOI:** 10.3389/fmed.2022.834887

**Published:** 2022-07-14

**Authors:** Sarah Damanti, Marta Cilla, Maria Cilona, Aldo Fici, Aurora Merolla, Giacomo Pacioni, Rebecca De Lorenzo, Sabina Martinenghi, Giordano Vitali, Cristiano Magnaghi, Anna Fumagalli, Mario Gennaro Mazza, Francesco Benedetti, Moreno Tresoldi, Patrizia Rovere Querini

**Affiliations:** ^1^Unit of General Medicine and Advanced Care, Istituto di Ricovero e Cura a Carattere Scientifico (IRCCS) San Raffaele Institute, Milan, Italy; ^2^Faculty of Medicine and Surgery, Vita-Salute San Raffaele University, Milan, Italy; ^3^San Raffaele Diabetes Research Institute, IRCCS Ospedale San Raffaele, Milan, Italy; ^4^Department of Immunology, Transplantation and Infectious Diseases, IRCCS Ospedale San Raffaele, Milan, Italy; ^5^COVID Trial Unit, Department of Internal Medicine, IRCCS San Raffaele Institute, Milan, Italy; ^6^Psychiatry & Clinical Psychobiology, Division of Neuroscience, IRCCS Scientific Institute Ospedale San Raffaele, Milan, Italy

**Keywords:** frailty, COVID-19, long COVID-19 syndrome, older people, prevalence

## Abstract

**Background:**

A motley postacute symptomatology may develop after COVID-19, irrespective of the acute disease severity, age, and comorbidities. Frail individuals have reduced physiological reserves and manifested a worse COVID-19 course, during the acute setting. However, it is still unknown, whether frailty may subtend some long COVID-19 manifestations. We explored the prevalence of long COVID-19 disturbs in COVID-19 survivals.

**Methods:**

This was an observational study. Patients aged 65 years or older were followed-up 1, 3, and 6 months after hospitalization for COVID-19 pneumonia.

**Results:**

A total of 382 patients were enrolled. Frail patients were more malnourished (median Mini Nutritional Assessment Short Form score 8 vs. 9, *p* = 0.001), at higher risk of sarcopenia [median Strength, Assistance with walking, Rising from a chair, Climbing stairs, and Falls (SARC-F) score 3 vs. 1.5, *p* = 0.003], and manifested a worse physical performance [median Short Physical Performance Battery (SPPB) score 10 vs. 11, *p* = 0.0007] than robust individuals, after hospital discharge following severe acute respiratory syndrome coronavirus 2 (SARS-CoV-2) pneumonia. Frailty was significantly associated with: (i) confusion, as a presenting symptom of COVID-19 [odds ratio (OR) 77.84, 95% CI 4.23–1432.49, *p* = 0.003]; (ii) malnutrition (MNA-SF: adjusted B –5.63, 95% CI –8.39 to –2.87, *p* < 0.001), risk of sarcopenia (SARC-F: adjusted B 9.11, 95% CI 3.10–15.13, *p* = 0.003), impaired muscle performance (SPPB: B –3.47, 95% CI –6.33 to –0.61, *p* = 0.02), complaints in mobility (adjusted OR 1674200.27, 95% CI 4.52–619924741831.25, *p* = 0.03), in self-care (adjusted OR 553305.56, 95% CI 376.37–813413358.35, *p* < 0.001), and in performing usual activities of daily living (OR 71.57, 95% CI 2.87–1782.53, *p* = 0.009) at 1-month follow-up; (iii) dyspnea [modified Medical Research Council (mMRC): B 4.83, 95% CI 1.32–8.33, *p* = 0.007] and risk of sarcopenia (SARC-F: B 7.12, 95% CI 2.17–12.07, *p* = 0.005) at 3-month follow-up; and (iv) difficulties in self-care (OR 2746.89, 95% CI 6.44–1172310.83, *p* = 0.01) at the 6-month follow-up. In a subgroup of patients (78 individuals), the prevalence of frailty increased at the 1-month follow-up compared to baseline (*p* = 0.009).

**Conclusion:**

The precocious identification of frail COVID-19 survivors, who manifest more motor and respiratory complaints during the follow-up, could improve the long-term management of these COVID-19 sequelae.

## Background

Coronavirus disease 2019 (COVID-19) can have heterogeneous manifestations (1), but a more severe course is expected in older people, due to the combined effect of immune-aging and accrual of comorbidities over time (2–5). In addition, the exhaustion of physiological reserves in older people, usually known as frailty (6), augments the vulnerability to stressors and enhances the risk of developing negative health outcomes (7). Indeed, a higher lethality of COVID-19 has been demonstrated in frail patients in the acute setting (8).

Worldwide reports showed that between 57 and 76% of people infected by severe acute respiratory syndrome coronavirus 2 (SARS-CoV-2) develop persistent COVID-19 sequelae (9, 10) irrespectively of acute COVID-19 severity and age (9, 11–13). The manifestations of the postacute SARS-CoV-2 (PASC) syndrome can be: either residual symptoms and organ dysfunctions persisting after the acute infection or *de-novo* manifestations, which can develop after the resolution of COVID-19 (13, 14). Their course can be fluctuating, increasing, persisting, or relapsing. The Italian National Institute of Health (ISS) divides the PASC syndrome into two categories, according to its length: (i) persistent symptomatic COVID-19, when symptoms and signs last between 4 and 12 weeks after the acute infection and (ii) post-COVID-19 syndrome, when the manifestations prolong more than 12 weeks after the acute disease (15).

The clinical spectrum of the so-called “long COVID-19” is motley, including fatigue, muscle weakness, dyspnea, chest pain, cognitive impairment, depression, anxiety, and insomnia (10).

The etiology of the PASC syndrome has not been completely clarified yet, but it is presumably multifactorial (i.e., dysregulated immune response, endothelial injury) (16, 17) and an organic substratum seems to be present. MRI studies in patients with post-COVID-19, who suffered from either a mild or a severe SARS-CoV-2 infection, showed a multiorgan impairment inflammation, regional scarring, and ectopic fat deposition (18, 19).

An association between comorbidities (particularly obesity and psychiatric conditions) and impairment in recovering completely after SARS-CoV-2 infection has been reported (20). Indeed, the exhaustion of physiological reserves (*i.e.*, frailty) (7) could favor the persistence of COVID-19 complaints. Many COVID-19 survivors manifest sarcopenia (21) because of the intense catabolic stimuli, bed rest, weight loss, inadequate protein supply, and steroid therapies during a hospital stay. The persistence of sarcopenia after hospital discharge (22) represents a condition closely related to frailty (23) and may subtend some long COVID-19 manifestations (i.e., fatigue, myalgias) (6).

No study has evaluated so far, whether long COVID-19 symptoms varied according to the frailty status in patients recovering after hospitalization for SARS-CoV-2 infection.

We analyzed whether the prevalence of the PASC symptoms was different between frail and robust patients, after hospital discharge, following COVID-19 pneumonia. Moreover, we assessed the association between frailty and the PASC manifestations at 1-, 3-, and 6-month follow-ups after hospital discharge, through regression analyses. Finally, we analyzed the variations of the frailty status over time during the follow-up visits.

## Materials and Methods

This was a prospective observational study. We evaluated patients aged 65 years or older, who attended a dedicated post-COVID-19 outpatient clinic. These patients were previously hospitalized for SARS-CoV-2 pneumonia in the Internal Medicine Department of the San Raffaele University Hospital, Milan, Italy (24) and discharged alive. Visits took place 1, 3, and 6 months after hospital discharge from 5 November 2020 to 2 November 2021.

The present study was part of the COVID-BioB study (NCT04318366). COVID-BioB wanted to characterize patients with COVID-19 who were hospitalized for pneumonia, through the prospective collection of demographic, anthropometric, clinical, and laboratory data (25). The COVID-BioB protocol was approved by the San Raffaele University Hospital Ethics Committee (protocol no. 34/int/2020).

During the follow-up visits, the patients underwent a multidimensional evaluation consisting in anamnesis, medical examination, anthropometric measurements, screening for sarcopenia through the Strength, Assistance with walking, Rising from a chair, Climbing stairs, and Falls (SARC-F) questionnaire (26), assessment of muscle strength through the hand grip strength test (27), evaluation of muscle performance with the Short Physical Performance Battery (SPPB) test (28), screening for malnutrition with the Mini Nutritional Assessment Short Form (MNA-SF) questionnaire (29), evaluation of the quality of life through the EuroQol Group Health Questionnaire 5D-3L (30), and the Visual Analog Scale (VAS) for general health (31). Moreover, the number of persistent and *de-novo* COVID-19 symptoms and manifestations were assessed at each visit. Symptoms were self-reported by the patients during the follow-up visits or written *via* email to the attending physicians of the follow-up clinic. All the patients with *de-novo* symptoms underwent a nasal swab for detecting an eventual *de-novo* SARS-CoV-2 infection.

According to a predictive algorithm (Supplementary Material) generated from the data of the first wave of the COVID-19 pandemic, only patients with: (i) a respiratory rate >17 breaths/min plus, either a history of coronary heart disease, or the general VAS score <86 or a C-reactive protein at hospital admission >149 mg/l or (ii) patients aged 63 years or older with a respiratory rate <17 breaths/min, but a body mass index (BMI)=26 kg/m^2^, continued the follow-ups at 3 and 6 months.

Frailty was measured with the frailty index (FI) (2, 11) created by using the criteria proposed by Searle et al. (32, 33). The variables used to generate the FI encompass comorbidities, baseline assessment data, and blood test results. Each deficit included in the FI was scored 0 when absent and 1 when present. Thirty-one variables (Supplementary Table 1) were used to calculate the baseline FI and 37 variables were used to calculate the FI at 1-, 3-, and 6-month follow-up. Using more than 30 variables to calculate the FI indexes confers sufficient robustness. The FI scores above 0.25 were classified as indicative of frailty (34).

### Statistical Analyses

The baseline characteristics of the study population, such as the main aspects of the COVID-19 hospitalization, quality of life, nutritional aspects, muscle parameters, the number and type of COVID-19 onset, and persisting and *de-novo* symptoms and manifestations at 1, 3, and 6 months after hospital discharge, were described through descriptive statistics. Continuous variables were presented as mean and SD when normally distributed or with median and interquartile range (IQR), when data had a skewed distribution. Dichotomous variables were presented as number (N) and percentage (%). A comparison of the distribution of categorical and continuous variables between frail and robust patients was made through the chi-squared test for categorical variables and the Mann–Whitney *U* test for continuous variables.

The univariate linear, binary logistic, multinomial logit, and ordinal regression analyses were performed to explore the association between frailty (assessed through the FI) and the PASC manifestations at 1-, 3-, and 6-month follow-ups. Age- and sex-adjusted models were also run for the significant predictors of the univariate analyses. Regression models were also repeated considering only the subgroup of patients who attended all the three follow-up visits.

The variations of the FI over time were evaluated with the Friedman test and the changes in the frailty status during the follow-up were evaluated with the Cochran’s Q test.

All the statistical analyses were performed with SPSS version 25.0 (SPSS Incorporation, Chicago, Illinois, United States).

## Results

**FIGURE 1:**
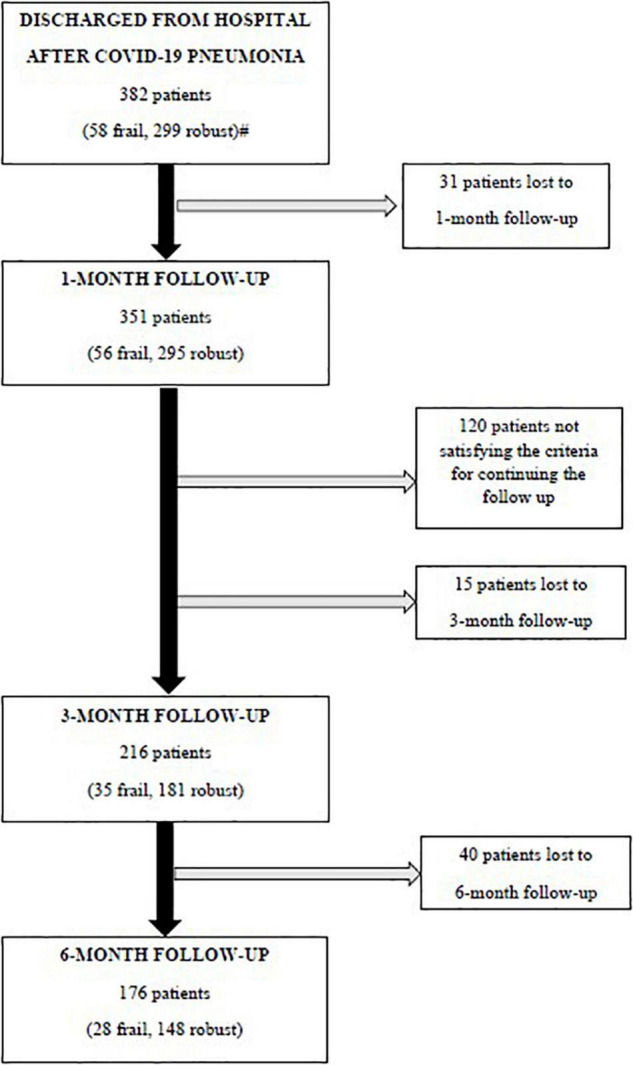
Flowchart of the follow-up visits. Criteria for continuing the follow-up: (i) a respiratory rate >17 breaths/min plus, either a history of coronary heart disease, or the general Visual Analog Scale (VAS) score <86 or a C-reactive protein at hospital admission >149 mg/l or (ii) patients aged 63 years or older with a respiratory rate <17 breaths/min, but a body mass index (BMI) ≥26 kg/m^2^, continued the follow-ups at 3 and 6 months. #Missing data for calculating the baseline frailty index in 25 patients.

**FIGURE 2:**
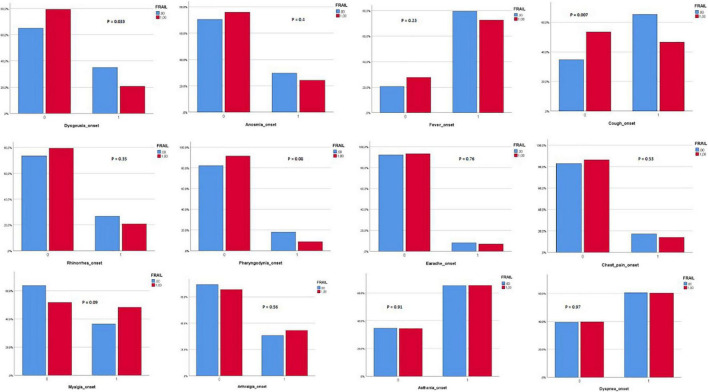
Length of hospital stay in frail and robust patients. Robust patients: Median: 14 days; 25^°^ percentile: 10 days; 75^°^ percentile: 23 days. Frail patients: Median: 18.5 days; 25^°^ percentile: 11 days; 75^°^ percentile: 30 days.

**FIGURE 3:**
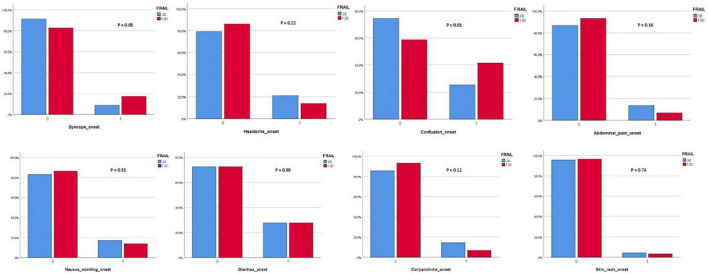
Days between symptoms onset and emergency room (ER) admission in frail and robust patients. Robust patients: Median: 7 days; 25^°^ percentile: 3 days; 75^°^ percentile: 10 days. Frail patients: Median: 5 days; 25^°^ percentile: 0.25 days; 75^°^ percentile: 9 days. ER, emergency room.

**FIGURE 4:**
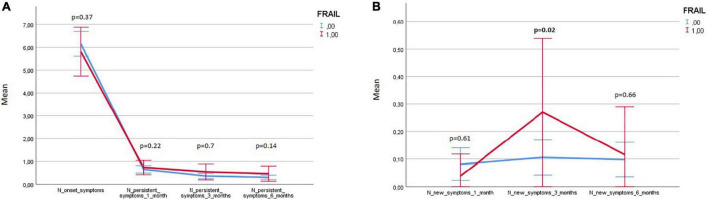
**(A,B)** Onset of symptoms in frail and robust patients.

Three hundred and eighty-two patients aged 65 years or older were hospitalized at the San Raffaele Hospital for SARS-CoV-2 pneumonia between 24 August 2020 and 6 June 2021 and were discharged alive. These patients were enrolled in the present study. The compliance to 1-month follow-ups was 98.3% (351 patients). Only 60.5% of the hospitalized patients satisfied the criteria for continuing the follow-up at 3 and 6 months. However, the compliance to the 3- and 6-month follow-up visits was reduced to 56.5 (216 patients) and 49.3% (176 patients) of the initial sample, respectively (Figure 1). Only 15.2% (58 individuals) of hospitalized patients could be considered frail at hospital admission, according to their FI score above 0.25. Frail patients were significantly (*p* < 0.001) older than robust individuals (median age 78 vs. 74 years) and had longer hospitalizations (median hospital stay: 18.5 vs. 14 days, *p* = 0.004), as shown in Figure 2. Moreover, they suffer from more comorbidities than robust patients (Table 1). Even if the median number of COVID-19 onset symptoms did not differ between frail and robust patients, frail individuals had a shorter latency, between symptoms onset, and emergency department (ED) admission (5 vs. 7 days, *p* = 0.03), as shown in Figure 3. In addition, frail patients manifested (41.4 vs. 25.6%, *p* = 0.01) confusion more frequently, as first symptom of COVID-19. Instead, dysgeusia (35 vs. 20.7%, *p* = 0.03) and cough (65.3 vs. 46.6%, *p* = 0.007) at COVID-19 onset were more frequently observed in robust individuals (Figures 4A,B and Table 1).

**TABLE 1:** Baseline and hospitalization characteristics of the study population^#^.

	Frail	Robust	p
	**(*N* = 58)**	**(*N* = 299)**	
Age	78 (IQR 72.8–82)	74 (IQR 69–80)	<0.001*
Males	41 (70.7%)	172 (57.5 %)	0.06**
Ethnicity			0.36**
*Caucasian*	57 (98.3%)	290 (97%)	
*Asiatic*	0 (0%)	2 (0.7%)	
*Latin American*	0 (0%)	6 (2%)	
*Black*	1 (1.7%)	1 (0.3%)	
BMI before COVID-19	27.4 (IQR 23.0–30.8)	27.3 (IQR 24.5–30.3)	0.76*
Frailty Index	0.29 (IQR 0.26–0.32)	0.13 (IQR 0.09–0.17)	<0.001*
Hypertension	47 (81%)	168 (56.2%)	<0.001**
Diabetes	27 (46.6%)	48 (16.1%)	<0.001**
Coronary heart disease	25 (43.1%)	46 (15.4%)	<0.001**
Heart failure	24 (41.4%)	15 (5%)	<0.001**
Arrhythmia	31 (53.4%)	42 (14.1%)	<0.001**
COPD/asthma/emphysema	16 (27.6%)	24 (8%)	<0.001**
Chronic kidney failure	16 (27.6%)	11 (3.7%)	<0.001**
Active neoplasia	4 (6.9%)	18 (6%)	0.80**
Rheumatic disease	6 (10.3%)	17 (5.7%)	0.19**
Arthrosis	8 (13.8%)	18 (6%)	0.04**
Osteoporosis	8 (13.8%)	19 (6.4%)	0.05**
Psychiatric illness	5 (8.6%)	25 (8.4%)	0.95**
Dementia	3 (5.2%)	7 (2.3%)	0.23**
Chronic neurological disease	8 (13.8%)	17 (5.7%)	0.03**
Hepatic disease	10 (17.2%)	24 (8%)	0.03**
Peptic ulcer	2 (3.4%)	12 (4%)	0.84**
Peripheral vascular disease	23 (39.7%)	35 (11.7%)	<0.001**
Cerebrovascular diseases	15 (25.9%)	18 (6%)	<0.001**
Length of hospital stay	18.5 (IQR 11–30)	14 (IQR 10–23)	0.004*
ICU stay	3 (5.2%)	13 (4.4%)	0.78**
NIV	18 (31%)	71 (23.9%)	0.25**
Thrombo-embolic events during hospital stay	2 (3.4%)	11 (3.7%)	0.93**
Arrhythmic events during hospitalization	3 (5.2%)	9 (3%)	0.41**
Days between symptoms’ onset ER admission	5 (IQR 0.25–9)	7 (IQR 3–10)	0.03*
COVID-19 onset symptoms			
*Dysgeusia*	12 (20.7%)	104 (35%)	0.033**
*Anosmia*	14 (24.1%)	88 (29.6%)	0.4**
*Fever*	42 (72.4%)	236 (79.5%)	0.23**
*Cough*	27 (46.6%)	194 (65.3%)	0.007**
*Rhinorrhea*	12 (20.7%)	79 (26.6%)	0.35**
*Pharyngodynia*	5 (8.6%)	53 (17.9%)	0.08**
*Earache*	4 (6.9%)	24 (8.1%)	0.76**
*Chestpain*	8 (13.8%)	51 (17.2%)	0.53**
*Myalgia*	28 (48.3%)	108 (36.4%)	0.09**
*Arthralgia*	20 (34.5%)	91 (30.6%)	0.56**
*Asthenia*	38 (65.5%)	193 (65%)	0.91**
*Dyspnea*	35 (60.3%)	180 (60.6%)	0.97**
*Syncope*	10 (17.2%)	26 (8.8%)	0.05**
*Headache*	8 (13.8%)	62 (20.9%)	0.22**
*Confusion*	24 (41.4%)	76 (25.6%)	0.01**
*Abdominalpain*	4 (6.9%)	40 (13.5%)	0.16**
*Nausea/vomiting*	8 (13.8%)	51 (17.2%)	0.53**
*Diarrhea*	16 (27.6%)	82 (27.6%)	0.99**
*Conjunctivitis*	4 (6.9%)	43 (14.5%)	0.12**
*Skin rash*	2 (3.4%)	13 (4.4%)	0.74**
Number of COVID-19 onset symptoms	5 (IQR 4–7)	6 (IQR 3–8)	0.37*

*BMI, Body Mass Index; ICU, Intensive Care Unit; NIV, Non Invasive Mechanical Ventilation; ER, Emergency Department.*

*^#^Missing data for calculating the baseline frailty index in 25 patients.*

*U Mann-Whitney test.

**Chi-Square test. Bold means statistically significant i.e. with p values < 0.05.

1 month after hospital discharge, frail patients appeared more malnourished (median MNA-SF score 8 vs. 9, *p* = 0.001), at higher risk of being sarcopenic (median SARC-F score 3 vs. 1.5, *p* = 0.003), and displayed a worse muscle performance (median SPPB score 10 vs. 11, *p* = 0.007) than robust individuals (Table 2).

**TABLE 2:** Nutritional and muscle characteristics, quality of life, and COVID-19 manifestations at the 1-month follow-up visits.

	Frail	Robust	p
	(*N* = 56)	(*N* = 295)	
BMI	26 (IQR 21.6–28.7)	26.1 (IQR 23.8–28.7)	0.46*
MNA-SF	8 (IQR 7–10)	9 (IQR 8–11)	0.001*
VAS	75 (IQR 50–80)	75 (IQR 60–90)	0.18*
mMRC	1 (IQR 0–3)	1 (IQR 0–2)	0.22*
SARC-F	3 (IQR 1–5)	1.5 (IQR 0–3)	0.003*
Hand Grip strength	18.9 (14.1–22.5)	19.2 (IQR 14.6–26.6)	0.24*
SPPB	10 (IQR 8–12)	11 (IQR 9–12)	0.007*
**EQ 5D-3L Mobility**			
*No problems*	25 (43.1%)	167 (55.9%)	0.04**
*Some problems*	23 (39.7%)	86 (28.8%)	
*Extreme problems*	2 (3.4%)	2 (0.7%)	
**EQ 5D-3L Self-care**			
*No problems*	37 (63.8%)	221 (73.9%)	0.03**
*Some problems*	7 (12.1%)	29 (9.7%)	
*Extreme problems*	6 (10.3%)	9 (3%)	
**EQ 5D-3L Usual activities**			
*No problems*	25 (43.1%)	170 (56.9%)	0.03**
*Some problems*	17 (29.3%)	71 (23.7%)	
*Extreme problems*	7 (12.1%)	14 (4.7%)	
**EQ 5D-3L Pain/Discomfort**			
*No problems*	24 (41.4%)	163 (54.5%)	0.12**
*Some problems*	24 (41.4%)	85 (28.4%)	
*Extreme problems*	2 (3.4%)	7 (2.3%)	
**EQ 5D-3L Anxiety/Depression**			
*No problems*	30 (51.7%)	141 (47.2%)	0.61**
*Some problems*	19 (32.8%)	105 (35.1%)	
*Extreme problems*	1 (1.7%)	12 (4%)	
**COVID-19 persistent symptoms**			
*Dysgeusia*	2 (3.4%)	12 (4%)	0.87**
*Anosmia*	1 (1.7%)	5 (1.7%)	0.97**
*Cough*	3 (5.2%)	20 (6.7%)	0.68**
*Rhinorrhea*	0 (0%)	3 (1%)	0.45**
*Pharyngodynia*	0 (0%)	1 (0.3%)	0.66**
*Chestpain*	1 (1.7%)	4 (1.3%)	0.81**
*Myalgia*	3 (5.2%)	9 (3%)	0.40**
*Arthralgia*	1 (1.7%)	3 (1%)	0.63**
*Asthenia*	6 (10.3%)	51 (17.1%)	0.21**
*Dyspnea*	9 (15.5%)	54 (18.1%)	0.66**
*Headache*	1 (1.7%)	5 (1.7%)	0.97**
*Confusion*	0 (0%)	1 (0.3%)	0.66**
*Other findings^§^*	0 (0%)	0 (0%)	n.a.
**Number of COVID-19 persistent symptoms**			
*0*	33 (56.9%)	181 (60.5%)	0.22*
*1*	20 (34.5%)	68 (22.7%)	
*2*	2 (3.4%)	25 (8.4%)	
*3*	1 (1.7%)	11 (3.7%)	
*4*	0 (0%)	4 (1.3%)	
**COVID-19 *de novo* symptoms**			
*Cough*	1 (1.7%)	0 (0%)	0.02**
*Chestpain*	0 (0%)	1 (0.3%)	0.66**
*Myalgia*	0 (0%)	5 (1.7%)	0.32**
*Arthralgia*	0 (0%)	1 (0.3%)	0.66**
*Asthenia*	1 (1.7%)	3 (1%)	0.63**
*Dyspnea*	0 (0%)	3 (1%)	0.45**
*Confusion*	0 (0%)	2 (0.7%)	0.53**
*Skin rash*	0 (0%)	1 (0.3%)	0.66**
*Deficits of short-term memory*	2 (3.4%)	6 (2%)	0.49**
*Alopecia*	0 (0%)	2 (0.7%)	0.53**
*Other findings#*	0 (0%)	0 (0%)	n.a.
**Number of COVID-19 *de novo* symptoms**			
*0*	52 (89.7%)	269 (90%)	0.61*
*1*	4 (6.9%)	16 (5.4%)	
2	0 (0%)	4 (1.3%)	

*BMI, Body Mass Index; MNA-SF, Mini Nutritional Assessment Short Form; VAS, Visual Analogue Scale; mMRC, Modified Medical Research Council Dyspnea Scale; SARC-F, Strength, Assistance with walking, Rising from a chair, Climbing stairs, and Falls questionnaire; SPPB, Short Physical Performance Battery.*

*^§^Abdominal pain, nausea/vomiting, diarrhoea, conjunctivitis, skin rash, earache, syncope, fever.*

*^#^Dysgeusia, anosmia, fever, headache.*

*U Mann-Whitney test.

**Chi-Square test. Bold means statistically significant i.e. with p values < 0.05.

Moreover, frail patients complained about more problems in mobility, self-care, and in performing usual activities. Instead, no difference was observed in the prevalence of COVID-19 persisting symptoms, but frail patients manifested a *de-novo* cough (1.7 vs. 0%, *p* = 0.02) more frequently. 3 months after hospital discharge (Table 3), frail patients manifested more *de-novo* COVID-19 symptoms (*p* = 0.02). In particular, frail patients manifested a *de-novo* dyspnea (3.4 vs. 0%, *p* = 0.001), myalgia (5.2 vs. 1%, *p* = 0.02), and fever (1.7 vs. 0%, *p* = 0.02) more frequently. Indeed, they complained about having more dyspnea [median modified Medical Research Council (mMRC) 2 vs. 0, *p* = 0.01]. Mobility complaints (*p* = 0.04) and risk of sarcopenia (median SARC-F score 3 vs. 1, *p* = 0.002) confirmed to be higher in frail patients at the 3-month follow-up too. At the 6-month follow-up (Table 4), frail patients kept complaining about a *de-novo* dyspnea (1.7 vs. 0%, *p* = 0.02) and about problems in mobility (*p* = 0.03) and self-care (*p* = 0.003) more frequently. In addition, their muscle performance (median SSPB score 11 vs. 12, *p* = 0.006) and general quality of life (VAS) were worse (median VAS score 65 vs. 75, *p* = 0.04).

**TABLE 3:** Nutritional and muscle characteristics, quality of life, and COVID-19 manifestations at the 3-month follow-up visits.

	Frail	Robust	p
	(*N* = 35)	(*N* = 181)	
BMI	26.9 (IQR 23.2–30.8)	27-3 (IQR 24.5–30)	0.36*
MNA-SF	13 (IQR 10–14)	14 (IQR 12–14)	0.24*
VAS	75 (IQR 50–90)	75 (IQR 70–85)	0.68*
mMRC	2 (IQR 0–3)	0 (IQR 0–2)	0.01*
SARC-F	3 (IQR 1–6)	1 (IQR 0–3)	0.002*
Hand Grip strength	19.8 (IQR 15.7–27.8)	21.7 (IQR 14.8–29.1)	0.71*
SPPB	11 (IQR 9–12)	12 (IQR 10–12)	0.08*
**EQ 5D-3L Mobility**			
*No problems*	19 (32.8%)	116 (38.8%)	0.04**
*Some problems*	14 (24.1%)	51 (17.1%)	
*Extreme problems*	1 (1.7%)	0 (0%)	
**EQ 5D-3L Self-care**			
*No problems*	23 (39.7%)	141 (47.2%)	0.24**
*Some problems*	7 (12.1%)	19 (6.4%)	
*Extreme problems*	1 (1.7%)	6 (3%)	
**EQ 5D-3L Usual activities**			
*No problems*	18 (31%)	120 (40.1%)	0.14**
*Some problems*	12 (20.7%)	37 (12.4%)	
*Extreme problems*	2 (3.4%)	6 (2%)	
**EQ 5D-3L Pain/Discomfort**			
*No problems*	12 (20.7%)	94 (31.4%)	0.06**
*Some problems*	20 (34.5%)	62 (20.7%)	
*Extreme problems*	1 (1.7%)	7 (2.3%)	
**EQ 5D-3L Anxiety/Depression**			
*No problems*	16 (27.6%)	90 (30.1%)	0.76**
*Some problems*	13 (22.4%)	62 (20.7%)	
*Extreme problems*	1 (1.7%)	10 (3.3%)	
**COVID-19 persistentsymptoms**			
*Dysgeusia*	2 (3.4%)	4 (1.3%)	0.25**
*Anosmia*	1 (1.7%)	2 (0.7%)	0.42**
*Cough*	1 (1.7%)	6 (2%)	0.89**
*Myalgia*	1 (1.7%)	6 (2%)	0.89**
*Arthralgia*	0 (0%)	1 (0.3%)	0.66**
*Asthenia*	3 (5.2%)	20 (6.7%)	0.67**
*Dyspnea*	7 (12.1%)	37 (12.4%)	0.96**
*Conjunctivitis*	0 (0%)	1 (0.3%)	0.66**
*Other findings^§^*	0 (0%)	0 (0%)	n.a.
**Number of COVID-19 persistent symptoms**			
*0*	25 (43.1%)	123 (41.1%)	0.7*
*1*	6 (10.3%)	45 (15.1%)	
*2*	3 (5.2%)	8 (2.7%)	
*3*	1 (1.7%)	3 (1%)	
*4*	0 (0%)	1 (0.3%)	
**COVID-19 *de novo* symptoms**			
*Fever*	1 (1.7%)	0 (0%)	0.02**
*Cough*	1 (1.7%)	1 (0.3%)	0.19**
*Myalgia*	3 (5.2%)	3 (1%)	0.02**
*Arthralgia*	0 (0%)	5 (1.7%)	0.32**
*Dyspnea*	2 (3.4%)	0 (0%)	0.001**
*Deficits of short term memory*	1 (1.7%)	3 (1%)	0.63**
*Alopecia*	1 (1.7%)	5 (1.7%)	0.97**
*Other findings^#^*	0 (0%)	0 (0%)	n.a.
**Number of COVID-19 *de novo* symptoms**			
*0*	25 (43.1%)	135 (45.2%)	0.02*
*1*	6 (10.3%)	10 (3.3%)	
*2*	0 (0%)	3 (1%)	
*3*	1 (1.7%)	0 (0%)	

*BMI, Body Mass Index; MNA-SF, Mini Nutritional Assessment Short Form; VAS, Visual Analogue Scale; mMRC, Modified Medical Research Council Dyspnea Scale; SARC-F, Strength, Assistance with walking, Rising from a chair, Climbing stairs, and Falls questionnaire; SPPB, Short Physical Performance Battery.*

*^§^Fever, rhinorrhea, pharyngodynia, earache, chest pain, syncope, headache, confusion, abdominal pain, nausea/vomiting, diarrhea, skin rash.*

*^#^Dysgeusia, anosmia, chest pain, asthenia, headache, confusion, skin rash.*

*U Mann-Whitney test.

**Chi-Square test. Bold means statistically significant i.e. with p values < 0.05.

**TABLE 4:** Nutritional and muscle characteristics, quality of life, and COVID-19 manifestations at the 6-month follow-up visits.

	Frail	Robust	p
	(*N* = 28)	(*N* = 148)	
BMI	25.1 (IQR 22.7–30.3)	27.6 (IQR 24.5–30.8)	0.09*
MNA-SF	14 (IQR 11–14)	14 (IQR 12.7–14)	0.4*
VAS	65 (IQR 50 -85)	75 (IQR 65–85)	0.04*
mMRC	0 (IQR 0–3)	0 (IQR 0–1)	0.20*
SARC-F	2 (IQR 0.25–4)	1 (IQR 0–3)	0.07*
Hand Grip strength	22.4 (IQR 17.8–27.1)	22.2 (IQR 16.3–28.6)	0.85*
SPPB	11 (IQR 10–12)	12 (IQR 11–12)	0.006*
**EQ 5D-3L Mobility**			0.03**
*No problems*	14 (24.1%)	99 (33.1%)	
*Some problems*	13 (22.4%)	37 (12.4%)	
*Extreme problems*	0 (0%)	0 (0%)	
**EQ 5D-3L Self-care**			0.003**
*No problems*	16 (27.6%)	117 (39.1%)	
*Some problems*	10 (17.2%)	16 (5.4%)	
*Extreme problems*	0 (0%)	2 (0.7%)	
**EQ 5D-3L Usual activities**			0.26**
*No problems*	16 (27.6%)	100 (33.4%)	
*Some problems*	10 (17.2%)	30 (10%)	
*Extreme problems*	1 (1.7%)	4 (1.3%)	
**EQ 5D-3L Pain/Discomfort**			0.91**
*No problems*	13 (22.4%)	73 (24.4%)	
*Some problems*	12 (20.7%)	56 (18.7%)	
*Extreme problems*	1 (1.7%)	6 (2%)	
**EQ 5D-3L Anxiety/Depression**			0.65**
*No problems*	17 (29.3%)	78 (26.1%)	
*Some problems*	8 (13.8%)	50 (16.7%)	
*Extreme problems*	2 (3.4%)	7 (2.3%)	
**COVID-19 persistent symptoms**			
*Dysgeusia*	0 (0%)	1 (0.3%)	0.66**
*Anosmia*	0 (0%)	1 (0.3%)	0.66**
*Cough*	1 (1.7%)	3 (1%)	0.61**
*Rhinorrhea*	0 (0%)	1 (0.3%)	0.66**
*Myalgia*	2 (3.4%)	2 (0.7%)	0.06**
*Arthralgia*	0 (0%)	1 (0.3%)	0.66**
*Asthenia*	4 (6.9%)	16 (5.4%)	0.59**
*Dyspnea*	5 (8.6%)	18 (6%)	0.41**
*Confusion*	1 (1.7%)	1 (0.3%)	0.18**
*Conjunctivitis*	0 (0%)	1 (0.3%)	0.66**
*Other findings* ^§^	0 (0%)	0 (0%)	n.a.
Number of COVID-19 persistent symptoms			0.14*
0	19 (32.8%)	108 (36.1%)	
1	6 (%)	29 (9.7%)	
2	2 (3.4%)	8 (2.7%)	
3	1 (1.7%)	0 (0%)	
**COVID-19 *de novo* symptoms**			
*Fever*	0 (0%)	1 (0.3%)	0.66**
*Chestpain*	0 (0%)	2 (0.7%)	0.54**
*Myalgia*	0 (0%)	1 (0.3%)	0.66**
*Arthralgia*	0 (0%)	2 (0.7%)	0.54**
	**(*N* = 28)**	**(*N* = 148)**	
*Asthenia*	(1.7%)	1 (0.3%)	0.18**
*Dyspnea*	(1.7%)	0 (0%)	0.02**
*Deficits of short-term memory*	1 (1.7%)	1 (0.3%)	0.18**
*Alopecia*	0 (0%)	3 (1%)	0.45**
*Other findings^#^*	0 (0%)	0 (0%)	n.a.
Number of COVID-19 *de novo* symptoms			0.66*
*0*	26 (44.8%)	136 (45.5%)	
*1*	1 (1.7%)	8 (2.7%)	
*2*	1 (1.7%)	2 (0.7%)	

*BMI, Body Mass Index; MNA-SF, Mini Nutritional Assessment Short Form; VAS, Visual Analogue Scale; mMRC, Modified Medical Research Council Dyspnea Scale; SARC-F, Strength, Assistance with walking, Rising from a chair, Climbing stairs, and Falls questionnaire; SPPB, Short Physical Performance Battery.*

§*Fever, pharyngodynia, earache, chest pain, syncope, headache, abdominal pain, nausea/vomiting, diarrhoea, skin rash. #Dysgeusia, anosmia, rhinorrhea, pharyngodynia, earache, syncope, cough, headache, diarrhoea, conjunctivitis, confusion, skin rash.

*U Mann-Whitney test.

**Chi-Square test. Bold means statistically significant i.e. with p values < 0.05.

**FIGURE 5:**
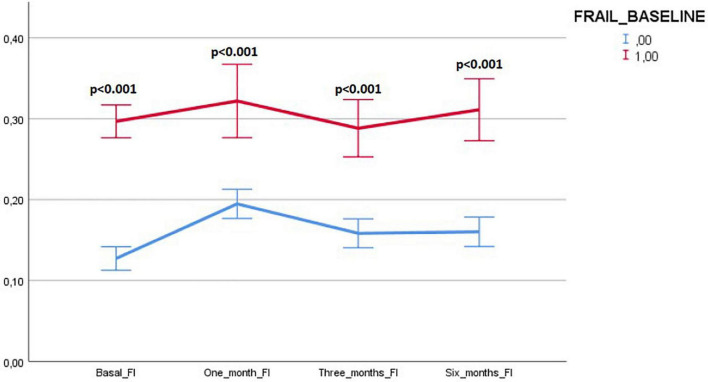
Variations in frail and robust patients of the number of persisting and *de-novo* long COVID-19 symptoms during the follow-up. Results expressed as means and 95% CIs.

Figure 5 illustrates the variations in frail and robust patients of the number of persistent and *de-novo* long COVID-19 symptoms during the follow-up.

In spite of the presence of *de-novo* symptoms, none of the patients manifesting these complaints at any time during the follow-up had SARS-CoV-2 reinfection.

The results of the univariate and age- and sex-adjusted regression analyses, which explored the association between frailty and the PASC manifestations during the follow-up, are given in Tables 5, 6, respectively. In the multivariate model, frailty was confirmed to be significantly associated with: (i) confusion, as a presenting symptom of COVID-19; (ii) malnutrition, risk of sarcopenia, impaired muscle performance, complaints in mobility, self-care, and in performing the usual activities of daily living at 1-month follow-up; (iii) dyspnea and risk of sarcopenia at 3-month follow-up; and (iv) difficulties in self-care at the 6-month follow-up.

**TABLE 5:** Results of the univariate regression models exploring the association between frailty and the PASC manifestations during the follow-up.

Confusion as initial symptom			
	**OR**	**95% C.I.**	**p**

F.I.	81.86	5.16–1297.53	0.002

**MNA-SF 1 month after hospital discharge**			

	**B**	**95% C.I.**	**p**

F.I.	−5.66	−8.31 to −3.02	<0.001

**SARCF 1 month after hospital discharge**			

	**B**	**95% C.I.**	**p**

F.I.	10.73	4.52–16.95	0.001

**SPPB 1 month after hospital discharge**			

	**B**	**95% C.I.**	**p**

F.I.	−6.1	−9.11 to −3.06	<0.001

**Mobility difficulties at 1 month-follow-up**			

	**OR**	**95% C.I.**	**p**

F.I.	591832.97	11.01–31807224594.91	0.17

**Difficulties in self-care at 1 month-follow-up**			

	**OR**	**95% C.I.**	**p**

F.I.	73951,392	179.51–30465008,684	<0.001

**Difficulties in usual activities at 1-month follow-up**			

	**OR**	**95% C.I.**	**p**

F.I.	20891,269	110.48–3950397.36	<0.001

***De novo* dyspnea at 3 month-follow-up**			

	**OR**	**95% C.I.**	**p**

F.I.	285293790,2	2.43–3,3442E16	0.04

**mMRC at 3 month-follow-up**			

	**B**	**95% C.I.**	**p**

F.I.	4.77	1.46–8.10	0.005

**Mobility difficulties at 3 month-follow-up**			

	**OR**	**95% C.I.**	**p**

F.I.	58.34	1.21–2820.51	0.04

**SARCF 3 months after hospital discharge**			

	**B**	**95% C.I.**	**p**

F.I.	9.28	4.06–14.49	0.001

**SPPB 6 months after hospital discharge**			

	**B**	**95% C.I.**	**p**

F.I.	−3.24	−6.29 to −0.18	0.04

**Mobility difficulties at 6 month-follow-up**			

	**OR**	**95% C.I.**	**p**

F.I.	144.43	1.86–11234.88	0.03

**Difficulties in self-care at 6 month-follow-up**			

	**OR**	**95% C.I.**	**p**

F.I.	3636.24	14.78–894614.89	0.03

*FI, Frailty Index; OR, Odds Ratio; C.I., Confidence Interval; MNA-SF, Mini Nutritional Assessment – Short Form; SARCF, Strength, Assistance with walking, Rising from a chair, Climbing stairs, and Falls questionnaire; SPPB, Short Physical Performance Battery; mMRC, modified Medical Research Council questionnaire.*

**TABLE 6:** Results of the age- and sex-adjusted regression models exploring the association between frailty and the PASC manifestations during the follow-up.

Confusion as initial symptom			
	**OR**	**95% C.I.**	**p**

F.I.	77.84	4.23–1432.49	0.003

**MNA-SF 1 month after hospital discharge**			

	**B**	**95% C.I.**	**p**

F.I.	−5.63	−8.39 to −2.87	< 0.001

**SARCF 1 month after hospital discharge**			

	**B**	**95% C.I.**	**p**

F.I.	9.11	3.10–15.13	0.003

**SPPB 1-month after hospital discharge**			

	**B**	**95% C.I.**	**p**

F.I.	−3.47	−6.33 to −0.61	0.02

**Mobility difficulties at 1 month-follow-up**			

	**OR**	**95% C.I.**	**p**

F.I.	1674200.27	4.52–619924741831.25	0.03

**Difficulties in self-care at 1 month-follow-up**			

	**OR**	**95% C.I.**	**p**

F.I.	553305.56	376.37–813413358.35	< 0.001

**Difficulties in usual activities at 1-month follow-up**			

	**OR**	**95% C.I.**	**p**

F.I.	71.57	2.87–1782.53	0.009

***De novo* dispnea at 3 month-follow-up**			

	**OR**	**95% C.I.**	**p**

F.I.	84066275.46	1.55–4,5527E15	0.045

**mMRC at 3 month-follow-up**			

	**B**	**95% C.I.**	**p**

F.I.	4.83	1.32–8.33	0.007

**SARCF 3 months after hospital discharge**			

	**B**	**95% C.I.**	**p**

F.I.	7.12	2.17–12.07	0.005

**Difficulties in self-care at 6 month-follow-up**			

	**OR**	**95% C.I.**	**p**

F.I.	2746.89	6.44–1172310.83	0.01

*FI, Frailty Index; OR, Odds Ratio; C.I., Confidence Interval; MNA-SF, Mini Nutritional Assessment – Short Form; SARCF, Strength, Assistance with walking, Rising from a chair, Climbing stairs, and Falls questionnaire; SPPB, Short Physical Performance Battery; mMRC, modified Medical Research Council questionnaire.*

In the subgroup analysis, including only patients who attended all the three follow-up visits, frailty was also associated with: (i) confusion, as a presenting symptom of COVID-19 [adjusted odds ratio (OR) 443.38, 95% CI 4.16–47237.39, *p* = 0.01]; (ii) malnutrition (MNA-SF: adjusted B –4.36, 95% CI –8.63 to –0.08, *p* = 0.046), risk of sarcopenia (SARC-F: adjusted B 7.1, 95% CI 1.75–12.42, *p* = 0.01), impaired muscle performance (SPPB: adjusted B –2.2, 95% CI –6.98 to –0.08, *p* = 0.046), complaints in mobility (adjusted OR 621.16, 95% CI 4.32–89415.18, *p* = 0.01), self-care (adjusted OR 1158.46, 95% CI 2.07–648668.62, *p* = 0.03), and in performing the usual activities of daily living (adjusted OR 12576.61, 95% CI 1.79–88327109.11, *p* = 0.037) at 1-month follow-up; (iii) dyspnea (mMRC: adjusted B 4.22, 95% CI 1.69–6.76, *p* = 0.001) and risk of sarcopenia (SARC-F: adjusted B 8.88, 95% CI 2.85–14.9, *p* = 0.004) at the 3-month follow-up; and (iv) difficulties in self-care (adjusted OR 2746.88, 95% CI 6.44–1172310.83, *p* = 0.01) at the 6-month follow-up.

**FIGURE 6:**
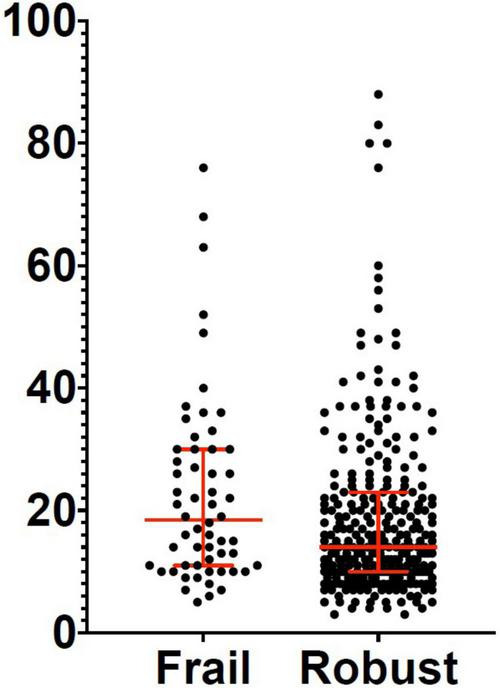
Length of hospital stay in frail and robust patients.

**FIGURE 7:**
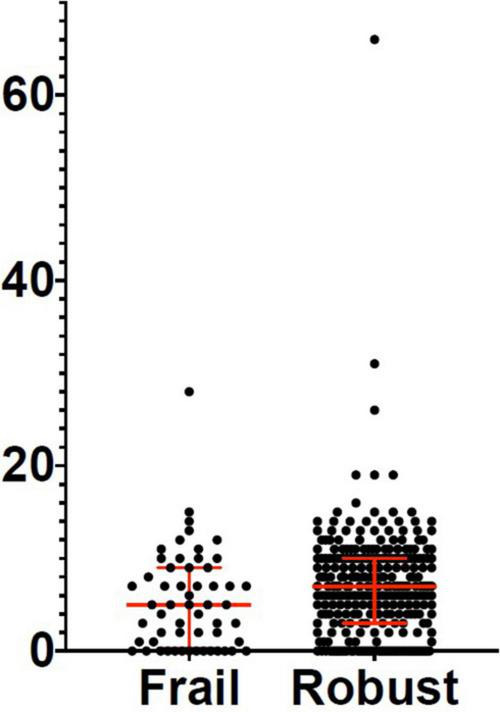
Interval between symptoms onset and ED admission in frail and robust patients.

Variations of the FI index and of the frailty status over time were assessed just in a subgroup of patients (78 subjects) for whom data for recalculating the FI during the follow-up visits were available. Tables 7, 8 illustrate the variations of the FI and the frailty status (defined as the FI >0.25) over time. Both the FI (*p* < 0.001) and the frailty status (*p* = 0.009) significantly changed during the follow-up (Figure 5 and Tables 7, 8). The highest values of the FI (median 0.2) and a number of frail individuals (23 people) were documented during the 1-month follow-up visits. Figures 6,7 illustrates the length of hospital stay in frail and robust patients (Figure 6) and the interval between COVID-19 symptoms onset and ED admission in frail and robust patients.

**TABLE 7:** Variations of the frailty index during the follow-up in a subgroup of 78 patients.

	Hospital admission	1-month follow up visit	3-month follow up visit	6-month follow up visit	p
**FI**	0.14 (IQR 0.10–0.23)	0.2 (IQR 0.15–0.27)	0.17 (IQR 0.11–0.23)	0.18 (IQR 0.14–0.25)	<0.001^§^

*FI, Frailty Index; IQR, Inter Quartile Range.*

*^§^Friedman test.*

**TABLE 8:** Variations of the frailty status during the follow-up in a subgroup of 78 patients.

	Robust	Frail	p
Hospital admission	61	17	**0.009^##^**
1-month follow up visit	55	23	
3-month follow up visit	64	14	
6-month follow up visit	61	17	

*Results are presented as number of patients in each category.*

*^##^Q Cochran test.*

*Bold means statistically significant i.e. with p values < 0.05.*

## Discussion

In this prospective observational study, we found that the latency between COVID-19 symptoms’ onset and emergency room (ER) admission was shorter in frail patients. Frail individuals had an atypical presentation of the SARS-CoV-2 infection with confusion, more frequently. The length of COVID-19 hospitalization was longer in frail patients. 1 month after hospital discharge, frail individuals were more malnourished, at higher risk of being sarcopenic, and manifested a worse physical performance than robust people. Indeed, frail patients complained more frequently about problems with mobility, self-care, and performing the usual activities of daily living. 3 months after hospital discharge, frail patients manifested more *de-novo* COVID-19 symptoms (in particular, dyspnea and myalgia), remained at higher risk of sarcopenia, and kept complaining about difficulties in mobility. Complaints in mobility and self-care and *de-novo* dyspnea were still present 6 months after hospital discharge. Indeed, muscle performance and general quality of life were found to be worse in frail patients 6 months after hospital discharge.

The regression analyses confirmed an association between frailty, malnutrition, risk of sarcopenia, mobility, usual activity impairments, and dyspnea during the follow-up.

In a subgroup analysis, the 1-month follow-up was the moment of a greater increase of the FI index over time when the highest number of frail individuals was detected.

Frail patients were older than robust individuals. This datum is consistent with the fact that the accumulation of health deficits and the decline in physiological reserve augment with aging, and as expected, the prevalence of frailty (7).

We found that the latency between symptoms’ onset and ER admission was shorter in frail patients. As far as we know, this is a new finding of our study that has never been reported yet. It is plausible that COVID-19 evolves more rapidly in people with reduced physiological reserves.

Frail patients presented COVID-19 in an atypical way, with confusion more frequently. Confusion can be a symptom of delirium. It has been demonstrated that delirium can be the only presenting sign of an infection (35) in older people. Recently, an observational American study has revealed that delirium was an ED-presenting symptom of COVID-19 in 28% of older people (36).

We detected longer hospitalization in frail patients compared to robust people. This finding is in line with the results of a large multicenter European study, (37) which showed that the duration of hospitalization was significantly longer in people with high values on the Clinical Frailty Scale (CFS) (≥5). Frail people need more time to recover from an acute infection and their hospitalization may more often be complicated by adverse intercurrent clinical events. Accordingly, it is reasonable to expect a more complicated postacute phase in frail COVID-19 survivors than in robust individuals. A recent study conducted by Jones et al. (38) in the primary care setting identified an association between frailty and long COVID-19 manifestations. However, this study included a heterogeneous population, with both patients, who only believed that they had COVID-19 (but without a clinical or test diagnosis) and patients with either a clinical or a test diagnosis of SARS-CoV-2 infection. Moreover, in this study, frailty was not assessed with a validated tool, but just with an answer to a question on the level of fitness.

We found that frail COVID-19 survivors were at greater risk of malnutrition and sarcopenia and complained about great mobility impairment and difficulties in performing the activities of daily living and in usual care, 1 month after hospital discharge. More mobility complaints persisted till the 6-month follow-up and became associated with a worse muscle performance during the 6-month visits after discharge. Our findings are in line with the results of Shinohara et al. (39) in Japanese community-dwelling older adults: frail patients complained about decreased subjective leg muscle strength.

It is known that acute sarcopenia, which is common among patients with COVID-19, can evolve into chronic sarcopenia (40), especially if people do not have adequate nutritional and protein supply and do not perform enough physical activity (41). Economic difficulties during the COVID-19 pandemic could have influenced the eating habits of older people, reducing their consumption of meat and other animal proteins. In addition, COVID-19 countermeasures had a negative impact on the time spent in performing physical activities (42).

Finally, the negative skeletal muscle manifestations could have been underpinned by increased levels of angiotensin II from the classical pathway and decreased angiotensin-converting enzyme 2 (ACE2)/Ang from the nonclassical pathway, leading to muscle wasting (5). Anyway, it should be underlined that we lacked data on muscle mass and function before hospital admissions. Thus, chronic sarcopenia could have been present in some patients also before SARS-CoV-2 infection.

A more frequent *de-novo* dyspnea in frail patients emerged during our COVID-19 survivors’ follow-ups. This result could be in line with the more common mobility complaints of frail patients, since both the manifestations could be underpinned by the presence of sarcopenia. Sarcopenia is particularly evident in the respiratory (43) and lower limb muscles. Sarcopenia of the respiratory muscles could impair the ability to produce appropriate tidal volumes (44) and to perform high force expulsive airway clearance maneuvers (45). Our group recently showed that reduction in respiratory muscle mass and quality was associated with extubation failure in COVID-19 critically ill patients (46).

The subgroup analyses demonstrated that frailty changed over time and COVID-19 survivors manifested a greater number of health deficits 1-month after hospital discharge.

Our findings are a bit different from the results of Lees et al. (47) who demonstrated in older people affected by influential an increased level of frailty from baseline to hospital admission, but a return to the baseline level of the frailty indexes 1 month after hospital discharge. We found instead a persistent deterioration of the frailty status in the first month after hospital discharge showing a negative impact of frailty on the recovery from the acute SARS-CoV-2 infection, which further increased the frailty of patients. However, also Lees et al. found that frailty hindered the recovery from the acute infection in flu patients (47).

Indeed, the inflammation caused by acute infections has systemic repercussions that can finally increase the frailty of the affected individuals. During infections, there is a progressive accumulation of unrepaired damages in tissues because the turnover of damaged macromolecules and organelles is inhibited (48). Moreover, both the inflammation and immobilization have catabolic effects on muscle mass contributing to physical function deterioration (49). Finally, infections can trigger other pathological conditions, which increase the level of frailty (50).

The first month after hospital discharge is a critical period for older people. Previous research revealed that after hospital discharge, many older people display impaired functional levels in spite of the resolution of the acute conditions, which lead to hospital admissions. These impairments tend to persist over time and increase patients’ risk of developing geriatric syndromes, novel disabilities, and hospital readmissions (51–56). In particular, the functional status 1 month after discharge was found to be associated with long-term outcomes (57). Adequate countermeasures should be introduced in the first 3 months after hospital discharge. These would favor a regain of the loss of functionality and would avoid a chronic course of the impairments (53).

Considering the symptom spectrum observed in this study in comparison to other respiratory diseases, we confirmed that frail patients more frequently manifested atypical symptoms (*i.e.*, confusion) (58). Indeed, in a previous study on the presentation of flu in a Canadian cohort, the flu diagnosis was missed in more than half of patients with laboratory-confirmed influenza, if clinicians based only on the influenza-like illness case definition (59).

Therefore, we suggest performing a follow-up in COVID-19 survivors 1 month after the acute disease resolution. Frail patients should continue this follow-up to evaluate whether their level of frailty changes over time. For example, frailty could be reassessed 3 and 6 months after the resolution of the acute disease. Moreover, in frail patients, adequate countermeasures to revert frailty should be suggested by physicians. Otherwise, frailty would become a self-perpetuating cycle.

Our study has the merit of having described a real-world cohort of old COVID-19 survivors, followed-up over 6 months, after hospital discharge after SARS-CoV-2 pneumonia. We first depicted the prevalence of persistent and *de-novo* long COVID-19 manifestations after hospital discharge, according to the frailty status. We used the FI to describe frailty. The FI is a macroscopic indicator of biological age and has the advantage of providing integrative information, (60, 61) important to predict adverse health outcomes (62). The quantification of the negative effects of the accumulation of deficits through the FI can estimate the individual’s biological age and risk profile and could be an innovative measure for assessing the risk of the PASC syndrome. This would have important therapeutic impacts. The identification of frail patients already at hospital admission would allow the prompt instauration of nutritional intervention and supervised physical exercise during a hospital stay. This could prevent and reduce the development of acute sarcopenia. Moreover, if these interventions were continued after hospital discharge, there would be also a reduction in the risk of chronicization of acute sarcopenia into chronic sarcopenia. Finally, frail individuals would benefit from a tighter follow-up that could promptly identify the PASC manifestations and ensure their adequate treatment.

However, some limits of this study are worth mentioning: the monocentric nature, the small sample, the fact that the nasal swab performed for evaluating eventual reinfections of SARS-CoV-2 was antigenic tests, the progressive reduction of the number of individuals involved in the follow-ups, and the lack of the evaluation of the frailty status over time in all the participants. Moreover, we did not have any information on patients’ muscle function before hospital admission. This prevented the identification of chronic vs. acute sarcopenia as a possible cause of some long COVID-19 manifestations.

The PASC syndrome has a negative impact on the social and clinical well-being of the patients. Therefore, it would be of paramount importance to adequately address the persistence of COVID-19 symptoms. The precocious identification of frail patients, who manifest more motor and respiratory complaints during the follow-up, would allow their addressing to *ad hoc* treatment and rehabilitative services, thus improving the long-term management of COVID-19 sequelae with a cost-effective and sustainable paradigm.

## Data Availability Statement

The raw data supporting the conclusions of this article will be made available by the authors, without undue reservation.

## Ethics Statement

The studies involving human participants were reviewed and approved by San Raffaele University Hospital Ethics Committee (protocol no. 34/int/2020). The patients/participants provided their written informed consent to participate in this study.

## Author Contributions

All authors made substantial contributions to the conception and design of the study, or acquisition of data, or analysis and interpretation of data, drafting the article or revising it critically for important intellectual content, and final approval of the version to be submitted.

## Conflict of Interest

The authors declare that the research was conducted in the absence of any commercial or financial relationships that could be construed as a potential conflict of interest.

## Publisher’s Note

All claims expressed in this article are solely those of the authors and do not necessarily represent those of their affiliated organizations, or those of the publisher, the editors and the reviewers. Any product that may be evaluated in this article, or claim that may be made by its manufacturer, is not guaranteed or endorsed by the publisher.
