# Supplementary figures and images for: Prevalence of Long COVID-19 Symptoms After Hospital Discharge in Frail and Robust Patients
Source: Front Med (Lausanne). 2022 Jul 14;9:834887. doi: 10.3389/fmed.2022.834887 (PMC9329529; doi:10.3389/fmed.2022.834887)

COVID-19 one-month follow-up visit after hospital discharge

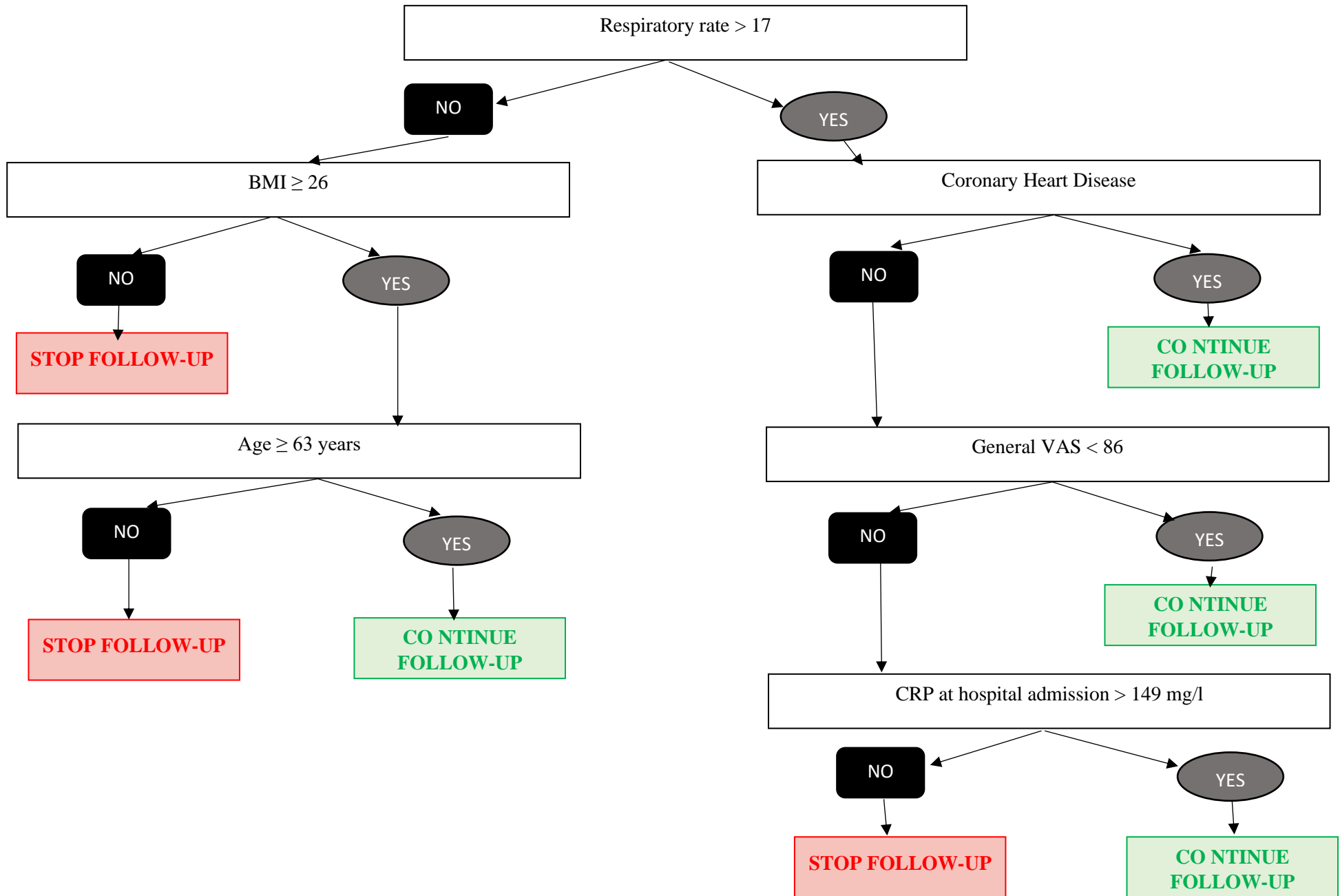

Supplement: Supplementary file 1 [file Data_Sheet_1.PDF]
